# Label-free microfluidic enrichment of ring-stage *Plasmodium falciparum*-infected red blood cells using non-inertial hydrodynamic lift

**DOI:** 10.1186/1475-2875-13-375

**Published:** 2014-09-20

**Authors:** Thomas M Geislinger, Sherwin Chan, Kirsten Moll, Achim Wixforth, Mats Wahlgren, Thomas Franke

**Affiliations:** Experimental Physics I, University of Augsburg, 86159 Augsburg, Germany; Department of Microbiology, Tumor and Cell Biology, Karolinska Institutet, Box 280, S-171 77 Stockholm, Sweden; Nanosystems Initiative Munich, Schellingstraße 4, 80799 Munich, Germany; Biomedical Engineering, University of Glasgow, Oakfield Avenue, G12 8LT Glasgow, Scotland, UK

**Keywords:** *Plasmodium falciparum*, Ring stage, Enrichment, Malaria, Microfluidic, Erythrocytes, Red blood cells, Lab-on-a-chip, Non-inertial lift

## Abstract

**Background:**

Understanding of malaria pathogenesis caused by *Plasmodium falciparum* has been greatly deepened since the introduction of *in vitro* culture system, but the lack of a method to enrich ring-stage parasites remains a technical challenge. Here, a novel way to enrich red blood cells containing parasites in the early ring stage is described and demonstrated.

**Methods:**

A simple, straight polydimethylsiloxane microchannel connected to two syringe pumps for sample injection and two height reservoirs for sample collection is used to enrich red blood cells containing parasites in the early ring stage (8-10 h p.i.). The separation is based on the non-inertial hydrodynamic lift effect, a repulsive cell-wall interaction that enables continuous and label-free separation with deformability as intrinsic marker.

**Results:**

The possibility to enrich red blood cells containing *P. falciparum* parasites at ring stage with a throughput of ~12,000 cells per hour and an average enrichment factor of 4.3 ± 0.5 is demonstrated.

**Conclusion:**

The method allows for the enrichment of red blood cells early after the invasion by *P. falciparum* parasites continuously and without any need to label the cells. The approach promises new possibilities to increase the sensitivity of downstream analyses like genomic- or diagnostic tests. The device can be produced as a cheap, disposable chip with mass production technologies and works without expensive peripheral equipment. This makes the approach interesting for the development of new devices for field use in resource poor settings and environments, e.g. with the aim to increase the sensitivity of microscope malaria diagnosis.

## Background

Malaria caused by *Plasmodium falciparum* is one of the most devastating parasitic diseases, taking an annual estimated death toll of about 627,000 [1]. While the parasite has a complex life cycle involving multiple stages in both the human host and the mosquito vector, only the asexual stage of the intra-erythrocytic developmental cycle (IDC) has been widely cultured *in vitro* due to the ease of manipulation and the clinical relevance [2]. The IDC stage of the parasite begins with the invasion of red blood cells (RBCs) by merozoites released from the hepatic stage, the intracellular parasite then matures progressively through ring stage, trophozoite stage and schizont stage in a roughly 48 hours cycle. At the end of each cycle, the mitotically-divided merozoites burst from the mature schizonts and reinitiate a new cycle by invading new RBCs [3]. The availability of the continuous *in vitro* culture system, combined with various methods for enrichment and synchronization of different stages within the IDC, has resulted in substantial progress made in understanding pathogenesis and biology of the parasite throughout the past decades.

Methods to synchronize and enrich the different asexual stages are necessary for many research strategies, for example in the preparation of antigens for immunological studies and in non-targeted proteomic studies, where contamination with host proteins would be undesirable [4]. All current synchronization and enrichment methods exploit the various physical and biochemical changes induced on the RBCs by the invading parasite. Physically, the invasion of the parasite does not result in a significant change in volume of the infected RBC (iRBC), but reduces the density compared to RBCs [5, 6]. The differential density enables the use of Percoll/ Ficoll density gradients to purify the mature trophozoite and schizont iRBCs [7, 8]. On the other hand, the parasite actively exports an abundance of proteins to create a new permeation pathway for metabolite transport and to facilitate immune evasion, and adherence of iRBCs to host cells. The exported proteins modify the iRBC membrane composition as well as alter the underlying cytoskeletal interactions resulting in increased cellular rigidity and susceptibility to osmotic pressure [9–11]. The sorbitol method of synchronization selects against mature stages by osmotic lysis of iRBCs, while flotation in gelatin utilizes the dampening of the sedimentation rate of iRBCs compared to RBCs in these substrates caused by the expression of knob protrusion on the *P. falciparum* infected RBC surface [12]. Likewise, the crystallization of haem into haemozoin makes it paramagnetic and is commonly employed for enrichment of mature stages through magnetic affinity purification [13]. Taking advantages of a combined synchronization and enrichment strategy, efficient enrichment of narrowly staged parasites can be achieved. Despite numerous methods to enrich the later stages, no current method appears to be efficient for enrichment of iRBCs containing the earlier ring stage from RBCs. At least not without compromising their structural integrity, since the former are only marginally different from uninfected RBCs in many physical or biochemical properties. While the ring stage parasites are metabolically less active than the late stages, ring stage parasites do undergo dynamic events of biological importance. Moreover, ring stage enrichment can be more readily applicable for studies involving parasite isolates from malaria patients, since the majority of parasites in peripheral blood will be of ring stage and often of low parasitaemia. Direct enrichment of ring stage parasites from these samples can avoid *in vitro* adaptation, as clinical isolates can sometimes be refractory to such manipulation.

Here, a novel way to enrich ring-stage iRBCs without labelling in a simple microfluidic device is demonstrated. The enrichment is based on a separation process driven by the non-inertial lift effect using the altered deformability of the iRBCs as intrinsic marker (non-inertial lift induced cell sorting, NILICS [14]).

## Methods

### Sample preparation

*P. falciparum* laboratory strain 3D7S8 [15] is cultivated according to standard methods and synchronize it using 5% sorbitol [15]. Freshly isolated RBCs are stored in blood bags and experiments are performed within one week after blood withdrawal. Prior to experiments, the stage of the parasite is identified by staining with acridine orange of fresh culture and subsequent direct fluorescence microscope analysis in combination with careful observation of the culture [15].

For the sorting experiments, the original parasite culture is centrifuged and 10 μl of the pellet are resuspended in 500 μl of a 10% w/w solution of dextran (MW: 400–500 kDa, Sigma Aldrich Inc.) in phosphate buffered saline (PBS) to a final haematocrit (Hct) of approximately 2%. The same dextran concentration is used for the sheath flow without adding cells to it.

### Setup and microchannel design

**Figure 1:**
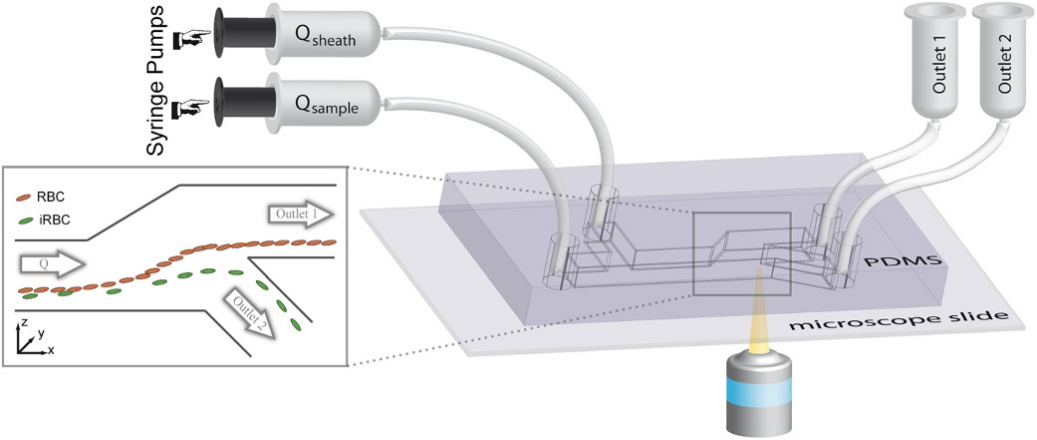
**Experimental setup.** The sheath flow Q_sheath_ and the sample flow Q_sample_ are driven by two separate syringe pumps. They are connected to the PDMS microchannel *via* PTFE tubes. The microchannel has a width w = 91 μm in y-direction for the whole device. The sample and the sheath flow inlet have a height h = 102 μm (in z-direction). The separation process occurs in the 20 mm long separation channel (height h = 102 μm) before the microchannel gently widens over a distance x = 400 μm to h = 301 μm. The bifurcation between outlet 1 and outlet 2 is at z = 50 μm and outlet 2 is connected at an angle of 49°. Both outlets have a height of h = 250 μm. The injected cells are focused to the lower wall before they flow through the separation channel and experience the non-inertial lift effect. The expansion shown in the inset increases the absolute height differences which facilitates sorting. Finally, the cells are collected in the height reservoirs connected to outlet 1 (waste) and outlet 2 (enriched sample). We observe the process using an inverted video microscope.

The setup for NILICS as depicted in Figure 1 consists of a single layer polydimethylsiloxane (PDMS) microchannel device fabricated using soft lithography [16]. The device features two inlets and two outlets. Each inlet is independently driven by a syringe pump (Harvard PHD2000, Harvard Apparatus) to provide separate control over the sheath flow Q_sheath_ and the sample flow Q_sample_, respectively. The Hamilton Gastight Syringes (Hamilton Bonaduz AG) containing the sample solutions are connected to the microchannel via polyfluoroethylene (PTFE) tubes. Outlets 1 and 2 are connected to height reservoirs that can be adjusted separately to control the hydrodynamic pressure in the respective outlet. The whole setup including syringes, tubes and the microchannel can be autoclaved. Here, the microchannel is simply flushed with the PBS/dextran solution directly before the experiments. The cells are observed optically using an inverted fluorescence microscope (Nikon Diaphot 300) equipped with a video camera (Orca 05G, Hamamatsu Photonics K.K.).

The microchannel is flushed just before the experiments with a 5 mg/ml bovine serum albumin (BSA) in water solution to prevent unspecific cell adhesion. The actual sorting then takes place at room temperature in a simple, straight, rectangular microchannel as shown in Figure 1. The sample solution containing the cells is injected into the microchannel with the flow rate Q_sample_ = 40 μl/h. The microchannel has a width w = 91 μm in y-direction (as denoted in the inset of Figure 1) for the whole device. The sample and the sheath flow inlet have a height h = 102 μm (in z-direction). The sample flow is focused towards the lower wall by a sheath flow with a typical flow rate of Q_sheath_ = 800 μl/h. The separation process occurs in the 20 mm long separation channel (height h = 102 μm) before the microchannel gently broadens from h = 102 μm over a distance x = 400 μm to h = 301 μm to amplify the absolute height difference between the cell populations. The cells follow the streamlines as they diverge, which facilitates sorting into outlet 1 or outlet 2 (h = 250 μm) which bifurcate 50 μm above the lower wall at an angle of 49°. The final height of the cell stream is adjusted by aligning the height reservoirs independently.

After the sorting experiments, the collected samples and the injected solution are centrifuged for 5 min at 2,000 rpm and washed thrice to remove the dextran. Then the samples are stained with Hoechst 33342 (Invitrogen, dilution 1:8,000 in PBS) for 30 min at RT, followed by two washing steps with PBS and are finally resuspended in 200 μl PBS. Flow cytometry (FACSVerse, BD Bioscience) is used to count the cells with the following routine: 250,000 total events are counted; alternatively, in samples with low cell numbers, data acquisition is carried out over 180 s. Data analysis is performed using the software FlowJo (Tree Star Inc.) and the parasitaemia
1

is determined.

The parasitaemia describes the quantitative content of iRBCs within a RBC population and is identical to the sample purity, which is the commonly used term to characterize the concentration of target cells speaking of cell sorting devices. The parasitaemia at the sample inlet and outlet 2 are used to calculate the enrichment factor
2

## Results

**Figure 2:**
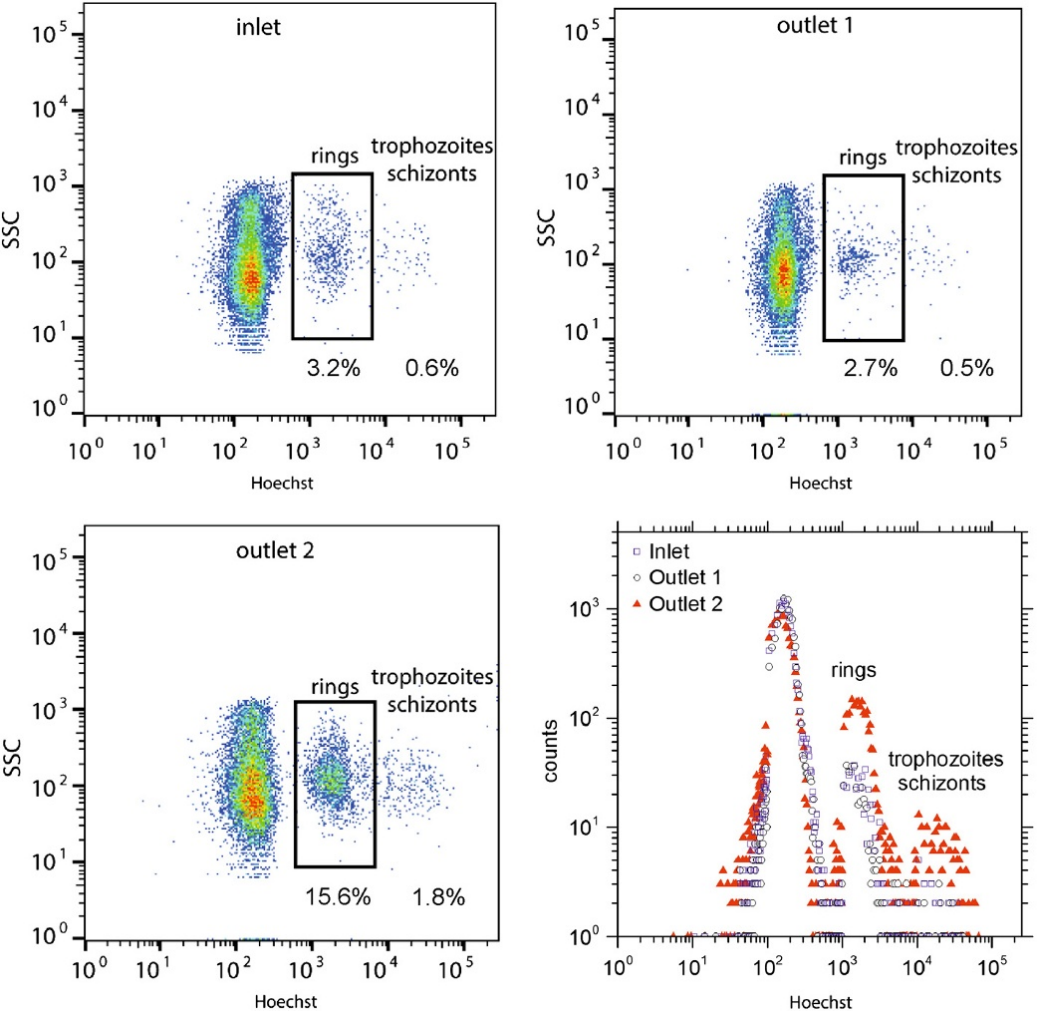
**Representative FACS analysis of the injected and collected samples.** The histogram shows the relative counts of all three samples.

Three independent sorting runs are conducted with mixtures of RBCs and iRBCs (8–10 h p.i.) from cell culture with different initial parasitaemia. Each run takes about one hour, corresponding to an injected sample volume of 40 μl. The height of the cell stream is adjusted using height reservoirs to sort approximately the one percent of the cells closest to the lower wall into outlet 2 while outlet 1 is used as waste. The collected samples are analysed using fluorescence activated cell sorting (FACS) to determine the parasitaemia at the sample inlet, outlet 1 and outlet 2. A typical result of the FACS analysis is shown in Figure 2. RBCs and iRBCs can be distinguished by staining with Hoechst, which labels DNA-containing cells. The more mature stages contain more DNA and thus – as is shown in Figure 2 - cause a higher fluorescence intensity. The separation in the phase space is clear and the cell populations are gated to determine the relative number of RBCs and iRBCs in the ring stage or in later stages (trophozoites/schizonts). The parasitaemia of each sample and cell population is summarized in Table 1. In the first run, there were not enough late stages apparent to set a proper gate. The histogram in Figure 2 illustrates the enrichment of iRBCs containing parasites at ring, trophozoite and schizont stage in outlet 2 compared to the inlet and outlet 1. If a pure ring stage iRBC solution is required for further experiments, iRBCs in the trophozoite and schizont stage can be lysed before the enrichment by a proper sorbitol treatment [15]. The counts are calculated as the total number of events in certain intervals of the fluorescence intensity. The width of the intervals is increased stepwise by an order of magnitude with every order of magnitude on the intensity axis. The enrichment factors given in Table 1 are calculated using the results of the FACS analysis in Eq. 2 and an average enrichment of iRBCs at ring stage by a factor of 4.3 ± 0,5 is found. In addition, to prove viability of the enriched cells, sorted ring-stages were returned to culture conditions and continued to be cultured for 48 hours. Parasites were optically controlled in the microscope after 24 h and 48 h and the development of the different parasite stages was as usual with no difference in duration of life cycle or invasion rates.

**Table 1:** **Parasitaemia (%) in samples from inlet, outlet 1 and outlet 2 and the enrichment for iRBCs in the ring and in later stages in three independent sorting runs, respectively**

Cell stage	Inlet	Outlet 1	Outlet 2	Enrichment (rings)	Enrichment (later stages)
Ring (~8 h p.i.)	1.6	1.6	6.1	3.8	---
Later stages	---	---	---	---	---
Ring (~10 h p.i.)	3.2	2.6	15.8	4.7	---
Later stages	0.6	0.5	1.8	---	3.0
Ring (~10 h p.i.)	3.4	2.1	15.7	4.5	---
Later Stages	0.6	0.5	2.0	---	3.3
	Average:			4.3 ± 0.5	3.2 ± 0.2

## Discussion

The separation principle presented here is based on the non-inertial lift effect. This effect occurs at very low Reynolds number flows (*Re* < 1, *Re* = *ρvD*/*μ*, with *ρ* = fluid density, *μ* = fluid viscosity, *v =* characteristic velocity*, D =* characteristic length) and is, therefore, a purely viscous phenomenon. The symmetry of the flow field in this strongly laminar regime is very high and the governing flow equation is reversible (Stokes flow) [17, 18]. Rigid spheres do not break this symmetry and, as a consequence, simply follow the flow streamlines. In contrast, soft and deformable objects, like RBCs, are able to generate an asymmetrical perturbation of the flow field if they have a predominant orientation with respect to the flow streamlines. To compensate for such a perturbation, the objects are pushed away from the adjacent wall towards the channel centerline [18–20]. *In vivo*, for RBCs in the microvascular system, the resulting rheological effects are known as the Fåhræus-Lindqvist effect [21, 22] and appear to be a direct consequence of the cell’s deformability. *In vitro,* the same effects being caused by the deformability of the RBCs can be used for cell separation.

**Figure 3:**
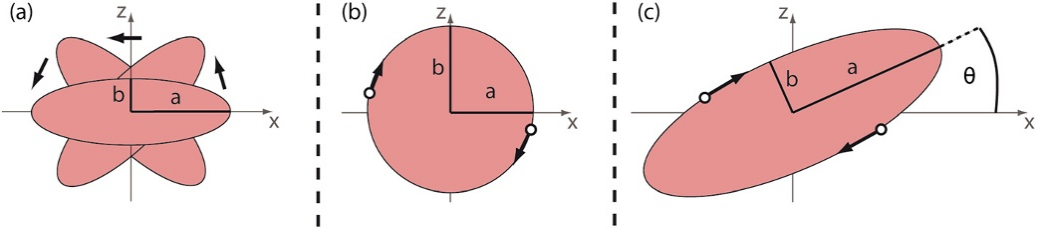
**Schematic drawing of the different regimes of motion exhibited by RBCs in shear flow. (a)** Tumbling at low shear stresses. The cell does not deform and rotates like a rigid body with a and b indicating the projection of the elliptical half axes in the paper plane. **(b)** At higher shear stress, the cell reorients itself and adopts the rolling motion. In this state of motion, the entire cell rotates. **(c)** At even higher shear stress, the RBCs reorient again and adopt the tank-treading state of motion. The cell is stretched and has a constant inclination angle θ with respect to the flow while the membrane rotates around the cytoplasm.

The deformation of RBCs subjected to external shear forces in the flow of a microchannel depends on three different parameters: the average cell viscosity η_in_ (membrane + cytoplasm, being a measure for the deformability), the fluid viscosity η_ext_ as well as the shear rate across the cell membrane [19, 23, 24]. The viscosities of the fluid and the RBCs are often combined using the viscosity ratio *λ* = *η*_*in*_/*η*_*ext*_. Examining theses parameters, several theoretical [25–27] and experimental [28–31] investigations revealed a complex phase landscape for the motion of capsules and vesicles in shear flow as a model system for RBCs. For low shear rates and high *λ*, RBCs exhibit a tumbling motion like solid particles, figuratively comparable to the flipping of a coin as illustrated schematically in Figure 3 [29]. With increasing shear force, the cells first reorient themselves and adopt a “rolling” state (Figure 3b). To evade membrane deformation, they align their disc axis perpendicular to the shear gradient leading to a rotation of the whole cell around that axis like for a rolling wheel [31]. Even higher shear stresses finally lead to a second reorientation of the RBCs to align their disc axis with the plane of the shear gradient (Figure 3c). The cell is stretched and the membrane starts to rotate around the cytoplasm. This so called tank treading (TT) motion is transferred to the cytoplasm which thus also starts to rotate and dissipate the shear stress while the cell adopts a constant inclination with respect to the flow [32, 33]. In the transition area from tumbling to tank treading, an intermittent regime exists in which the membrane is rotating but the inclination angle of the cell is not stationary. Instead, one observes a “swinging” around its steady state value [34]. A physiological consequence of the TT motion is the release of ATP [35] and, since TT and swinging lead to a predominant orientation of the RBCs, the migration of the cells towards the microchannel center caused by the non-inertial lift effect.

The magnitude of the wall induced non-inertial lift velocity depends on several parameters. Assuming the cell to be sufficiently far away from the wall (*z* > > *R*), the lift velocity *v*_*l*_ of TT vesicles can be described by
3

where  with the elliptical half axes *a*_*1*_*, a*_*2*_ and *a*_*3*_ denotes the effective vesicle radius and *U(λ,s*_*1*_*,s*_*2*_*)* the dimensionless drift velocity [19]. This theoretical prediction was found to be in good agreement to experimental results for vesicles [36] as well as RBCs and blood platelets [37]. The explicit dependence of the dimensionless drift *U(λ,s*_*1*_*,s*_*2*_*)* on the viscosity contrast *λ* = *η*_*in*_/*η*_*ext*_ and the shape parameters *s*_*i*_ = *a*_*i*_/*a*_3_ is quite complex. However, generally *U(λ,s*_*1*_*,s*_*2*_*)* decreases monotonically for increasing *λ,* and thus decreases monotonically with decreasing deformability of the RBCs [19].

The invasion of a malaria parasite and its growth within the RBC over several stages clearly affects the physical and chemical properties of the host cell [38]. As the parasite matures, the iRBCs for example become progressively spherical by losing parts of their membrane while keeping an almost constant volume [6]. The deformability changes in the later stages are dominated by the growing parasite itself and the ongoing crystallization of haem into haemozoin [38]. In contrast, parasite derived proteins that alter the iRBC membrane play the major role in the ring stage of the parasite development. Early after the invasion, the ring-infected erythrocyte surface antigen (RESA) is introduced into the spectrin network of the iRBCs cytoskeleton [39]. RESA stabilizes the spectrin tetramers to suppress further parasite invasions [40]. This plays the dominant role in the deformability changes in the early ring stage, while the influence of the parasite itself is faint at this point of development [41].

Micropipette aspiration measurements [41], a microfluidic device measuring the pressure needed to squeeze iRBCs through well-defined funnel constrictions [42] and diffraction phase microscopy [43] all find an approximately two-fold increase of the iRBCs’ stiffness in the ring stage compared to uninfected control cells. This change in deformability causes a significantly altered dynamic response of the iRBCs to shear stress. The fraction of TT iRBCs was found to be smaller (38% - 72%) at physiological shear stresses of 1–8 Pa than it is for the uninfected control cells (80% - 92%) at the same shear level [10]. This impaired deformability influences the strength of the non-inertial lift effect as predicted by Eq. 3. The decreased deformability leads to a decrease of the dimensionless drift *U(λ,s*_*1*_*,s*_*2*_*)* which results in a lower adopted height of iRBCs at the end of our microchannel compared to RBCs.

This difference of mechanical properties is used to continuously and label-free enrich iRBCs in the early ring stage (8–10 h p.i.) by employing the non-inertial lift effect. Here, continuously means that cells are processed in a continuous flow. Compared to the only available routine method for the enrichment of ring-staged iRBCs, which relies on picking single cells with the micromanipulator, a high throughput method is presented with actually ~ 12,000 sorted cells per hour. Additionally, this label-free approach avoids staining, enabling shorter laboratory protocols and providing unaltered cells for further analyses.

Apart from single cell micromanipulation, the only available method to enrich ring-staged iRBCs is another microfluidic approach based on the margination effect [44]. This term describes the lateral displacement of stiffer cells towards the channel walls by multiple collisions with inward migrating, more deformable cells. This collective effect works only at high haematocrit and is well-known from the displacement of white blood cells by RBCs in the microvasculature. Exploiting margination for the enrichment of malaria infected RBCs requires haematocrits of Hct > 40% [44]. This vast amount of RBCs in the microchannel leads to many ill-sorted cells and thus prevents the achievement of a high parasitaemia in the collected samples. Additionally, the device has only been demonstrated to enable an enrichment of ~ two-fold for iRBCs containing trophozoites or schizonts [44]. Although it has been demonstrated to enable sorting of iRBCs also in the ring stage, neither the precise parasite age past invasion nor a value for the corresponding enrichment is mentioned [44]. In contrast, our approach does not presume high haematocrits and is able to enrich even early ring stages (8 – 10 h p.i.) with an average enrichment factor of E = 4.3 ± 0.5. Thus, as a direct consequence of the low hematocrit, the parasitaemia of the collected samples is comparably high with our device, facilitating further enrichment runs and downstream analyses. Although the later stages are even less deformable and should thus experience an even weaker lift effect, they cannot be enriched as efficiently as ring stages, as reflected by the average enrichment of E = 3.2 ± 0.2 (data summarized in Table 1). This apparent contradiction is solved if the applied shear stress is taken into account. In the microchannel at the given flow conditions, the cells experience shear stresses larger than 30 Pa. This value is comparable to the physiological shear stress in the human spleen [45] and thus in a range, in which trophozoites and schizonts are typically lysed. Hence, many of the injected late stage iRBCs are lysed while passing the microchannel. Thus, the enrichment is worse for late stages than for rings, which withstand such shear stresses.

The enrichment could be even improved if the cell stream was adjusted to sort less than one percent of the cells into outlet 2. This, however, would further decrease the number of collected cells. However, microfluidic integration offers standard methods to increase throughput and efficiency by combination of several single devices in serial or in parallel [46]. Using multiple microchannels in series should strongly increase the enrichment effect while parallelization of several microchannels can increase the throughput of the device and thus shorten the processing times for larger sample volumes. As iRBCs in the ring stage are even less deformable at febrile temperatures [41] running the process at such elevated temperatures would in fact complicate the setup a little bit but should improve the enrichment. Also the enrichment from patient isolates is assumed to show even better results than the enrichment from cultured samples since the deformability of RBCs is already impaired by storage and the cell culture procedure. To this end, it is not yet clear if the alterations of deformability during storage and cell culture are uniform for infected and uninfected cells.

## Conclusions

In this study, passive, continuous and label-free enrichment of viable iRBCs containing *P. falciparum* parasites in the ring stage is demonstrated. For this purpose, a simple microfluidic device based on the non-inertial lift effect to drive the separation (non-inertial lift induced cell sorting, NILICS) is designed and employed. The non-inertial lift effect is sensitive to deformability changes of cells, which in turn act as intrinsic markers for the separation. Such deformability alterations are well known to accompany the invasion of RBCs by *P. falciparum* parasites and its intra-erythrocytic developmental cycle. Thus, the cell populations are separated by exploiting the different strength of the lift effect on RBCs and iRBCs. An average enrichment factor of E = 4.3 ± 0.5 is found for iRBCs in the early ring stage (8 - 10 h p.i.), outnumbering the only currently available microfluidic method by far. Compared to single cell picking with the micromanipulator, representing the only routine method for the enrichment of ring-staged *P. falciparum* infected RBCs, a high throughput method is presented, being able to process about 12,000 cells per hour. The method is suitable for laboratory use to improve the sensitivity of downstream analyses. Additionally, it can serve as a basis for the development of new devices for use in resource-poor settings and environments, e.g. with the aim to increase the sensitivity of microscope malaria diagnosis.

## Abbreviations

BSA: Bovine serum albumin
FACS: Fluorescence activated cell sorting
Hct: Haematocrit
IDC: Intra-erythrocytic developmental cycle
MW: Molecular weight
NILICS: Non-inertial lift induced cell sorting
PBS: Polyphosphate buffered saline
PDMS: Polydimethylsiloxane
PTFE: Polytetrafluorethylene
TT: Tank treading.
